# What is the optimal cardiothoracic surgery residency model?

**DOI:** 10.1016/j.xjon.2021.01.012

**Published:** 2021-03-04

**Authors:** Craig J. Baker

**Affiliations:** Department of Surgery, Cardiovascular and Thoracic Institute, University of Southern California Keck School of Medicine, Los Angeles, Calif

**Keywords:** I-6, integrated, thoracic surgery, education, traditional, thoracic residency

**Figure:**
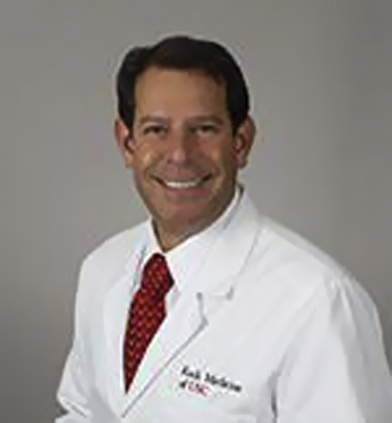
Craig J. Baker, MD, FACS

**Central Message**

Integrated programs developed during a time of heightened stress in our specialty offer numerous benefits compared with traditional programs. More programs should consider an integrated structure.

See Commentaries on pages 302, 304, and 306.

**Feature Editor's Introduction—**In the 21st century, we have witnessed great advancements in a wide range of fields, such as big data, computer sciences, and pharma. While these other fields have evolved and transformed, why do we insist on training future cardiothoracic surgeons using 100-year-old methods? The field of cardiothoracic surgery has experienced a great deal of change since its inception, yet it is only in the last 15 years that the training and educational paradigm in this field has begun to shift.

Innovation is what drives continued quality in almost any discipline. As Dr Baker notes, “a culture of educational excellence” should be the bedrock of any training program, big or small, traditional or integrated. Therefore, let us innovate! Let us take the methods that have produced highly capable and qualified surgeons in the past and adapt them to the new realities of modern surgical training needs. Let us dive into a new training paradigm with a progressive mindset, which not only honors our rich surgical traditions, but also reaffirms our commitment to better serve the needs of modern cardiothoracic surgical training.

As with any new approach, known and unforeseen challenges will surface. Attrition, inexperience of educators, and inexperience of trainees are all opportunities for learning. Providing a modern educational environment can help ensure that we will meet and overcome these and other future challenges, as generations before us have done.

**Rafael Durán, MD, and Nahush A. Mokadam, MD**

As the saying goes, “culture eats strategy for breakfast.” In writing this article, I thought it important to state the obvious. The culture of education within any given training program is vastly more important than the type of training paradigm offered at any given institution. True mentorship of trainees and commitment to educational excellence can produce talented physicians, surgeons, and academic leaders. To this extent, at The University of Southern California, we have an I-6 training program and a traditional 3-year independent training program. I have graduated residents from both pathways who I would trust as partners and who I would let operate on my loved ones. The question at hand and of this invited expert opinion presumes a culture of educational excellence. What then is the optimal cardiothoracic surgery residency model?

The American Board of Thoracic Surgery currently recognizes 4 pathways to certification.^1^

Pathway 1 requires completion of an Accreditation Council for Graduate Medical Education (ACGME)-approved general surgery residency or a 4/3 approved general surgery/thoracic surgery joint training program followed by the successful completion of an ACGME-approved thoracic surgery residency.
Pathway 2 requires successful completion of a full 5-year residency in general surgery, cardiac surgery or vascular surgery accredited by the Royal College of Physicians and Surgeons of Canada, followed by the successful completion of an ACGME-approved thoracic surgery residency.
Pathway 3 requires completion of a 6-year integrated thoracic surgery residency developed along guidelines established by the Thoracic Surgery Directors Association and approved by the ACGME.
Pathway 4 requires completion of an ACGME-approved 5-year vascular surgery residency that can lead to primary certification by the ABS, followed by the successful completion of an ACGME-approved thoracic surgery residency.

For the purposes of this article, I divide current training models into “traditional” and “integrated” pathways. Pathways 1 and 2 are considered traditional. The 4/3 paradigm, although differing among institutions, essentially allows for 2 to 3 years of thoracic surgery training following 4 years of general surgery training and functions very similarly to traditional thoracic surgery training. Pathway 3 (I-6 or integrated) is discussed extensively in this article. Pathway 4 permits matriculation into a traditional thoracic surgery residency after completion of an integrated vascular surgery residency. This likely merits a unique discussion but allows for significant exposure to cardiovascular disease during the first 3 years of training and thus is considered an integrated pathway.

In his landmark presidential address, “An Endangered Species,” delivered at the 35th annual meeting of the Western Thoracic Surgical Association in 2009, Dr David Fullerton outlined the monumental challenges facing thoracic surgery.^2^ He stated that “the only way by which the specialty of thoracic surgery can effectively change its phenotype is through the educational paradigms of thoracic surgical education.” Dr Fullerton acknowledged the first I-6 program developed at Stanford and 3 other programs the followed suit. I recall sitting in the audience as a newly inducted member of the Western Thoracic Surgical Association and acknowledging this call to arms, as I had been recently appointed program director at the University of Southern California (USC).

**Figure 1:**
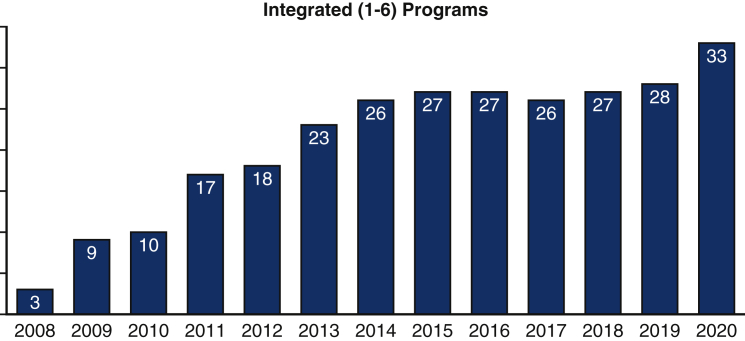
Number of US integrated thoracic surgery residency programs, 2008 to 2020. Data are from the Accreditation Council for Graduate Medical Education.

Over the next 5 years, numerous programs, including ours, initiated I-6 programs increasing the total number of programs to 26 by 2014. Since that time, there has been a plateau, with very few programs coming online (Figure 1).

When I initially proposed our I-6 program at USC, I was confronted with the usual skepticism: lack of maturity, surgical inexperience, reluctance to teach younger learners, and so on. Regardless, we pushed forward enthusiastically and accepted our first 2 residents in 2012. Exceeding all expectations, they both graduated in 2018 and were hired as faculty at USC after completing congenital training.

There are numerous advantages to the I-6 pathway. Our specialty has changed vastly, with an explosion of new technology and innovation. Six years of clinical instruction in thoracic surgery allows early exposure to physiology, surgical technique, and technology platforms that are unique to our specialty and no longer emphasized in general surgery. An opportunity to structure rotations in advanced imaging, perfusion, pulmonology, and endovascular skills more adequately prepares integrated residents to achieve comprehensive training and be true specialists in the treatment of thoracic diseases. One important benefit of the integrated pathway is the prolonged longitudinal relationship and mentorship that develops between faculty and trainees over 6 years. In my opinion, residents entering their fourth year of I-6 training are significantly more prepared compared with traditional residents entering a PGY 6 years after completing general surgery having mastered many nonapplicable skills.

The real advantage of an integrated program is not to shorten training, but rather to develop a truly integrated platform. This means spending substantial time on specialty-specific rotations during the first 3 years of training. I would argue that spending 1 or 2 months on cardiac rotations during the first 3 years does not define a true integrated curriculum. An integrated program should offer significant time on cardiothoracic rotations while maintaining those components of general surgery considered vital for any practicing surgeon. These would include rotations in critical care, acute care surgery, and gastrointestinal surgery, including important elements of colorectal and hepatobiliary surgery, but not the ability to perform advanced procedures in these specialized areas.

**Figure 2:**
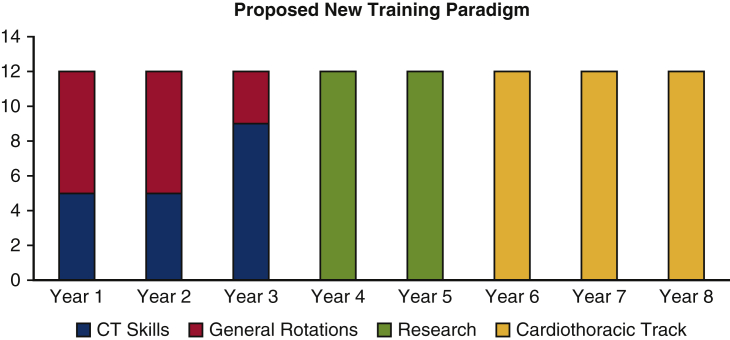
Thoracic surgical education in a changing paradigm.

A decade after Dr Fullerton's “call to arms,” Dr Vaughn Starnes delivered his powerful presidential address, “Thoracic Surgical Education in a Changing Paradigm,” at the 100th meeting of the AATS.^3^ Offering a comprehensive review of surgical education in the United States, Dr Starnes acknowledged that “although our field of thoracic surgery has expanded exponentially, our training paradigm has remained rather stagnant.” Is it possible that the same model of 2 to 3 years of thoracic surgery following general surgery training proposed in 1936 remains the optimal model of training? Dr Starnes proposed a 6-year training program with an optional 1 year or 2 years of focused research. He defined an escalating amount of thoracic surgery rotations during the first 3 years. He called for no less than 5 months of cardiovascular and thoracic rotation beginning in years 1 and 2. Year 3 would include 9 months of cardiothoracic exposure with full specialty immersion by year 4 (Figure 2). This proposed 6-year curriculum could be easily structured within the integrated (I-6) model approved by the ABTS and adopted by 33 programs around the United States.

Another challenge to our specialty is how to train in advanced structural heart, endovascular therapies, and potential emerging technologies. I believe it would be unfortunate to mandate subspecialty certification in these areas after completion of a traditional 2- or 3-year independent program when there would be ample time within an well-structured integrated 6-year program to offer training in these areas. We have recently introduced 8 months of elective time spanning the final 4 years of training for trainees wishing to pursue these skills. As case requirements become defined in these areas, programs should be able to refine their long-standing curricula to incorporate these skills during residency training. Another option would be to consolidate elective rotations into the final year to allow a full year of specialty training before entering practice. This also could also be structured as a seventh year at the home institution or an away site but should be considered part of the thoracic surgery residency. Fellowship training after thoracic residency may be important to gain experience in novel procedures, but we should alter our training programs to include core competence in emerging techniques as they become increasingly common components of everyday practice.

Characteristics of an ideal training program include:

• Six to 7 years of clinical training
• Substantial (5-6 months per year) early exposure to thoracic and cardiovascular surgery during the first 3 years of residency
• Early rotations in core general surgery principles and acute care surgery
• Optional 1 to 2 years of dedicated research time in the middle of training
• Eight to 12 months of elective time during the final 3 years for specialized training or possibly taken entirely during the sixth or seventh year of training
• Flexibility to allow rotations at other institutions if needed.

Integrated programs were born at a time of significant stress on our specialty. We were not attracting the best and brightest, board passage rates were declining, and interest in thoracic surgery seemed to be diminishing. Programs that established I-6 pathways, including ours, received applications from some of the most motivated and accomplished medical students from around the country. This trend certainly continues today. Although I am proud of my own thoracic surgical training at USC, I will attest that the residents we are graduating today are more prepared and qualified for independent practice than those of years past. Certainly this can be attributed to the evolving maturity and educational culture of our training program in general, but as I mentioned earlier, I-6 trainees beginning their last 3 years are better equipped, having already mastered early skills of sternotomy, internal mammary artery takedown, cannulation, and performance of basic thoracic surgery cases as the primary surgeon.

We have witnessed a renewed interest in thoracic surgery over the last decade, once again attracting he best and the brightest. Although this trend is exciting, and our training programs once again have ample applicants to choose from, I believe this has diminished the impetus for program directors to adopt an integrated pathway.

In the near future, there likely will not be a single pathway that fits all. Matching into an integrated thoracic surgery program selects out medical students who have made an early career decision to pursue our great specialty. There will continue to be outstanding trainees who matched into general surgery and decide to pursue thoracic surgery. As a specialty, we want the opportunity to train these candidates. This is one of the reasons I continue to offer both pathways. Ideally, there would be a mechanism for general surgery residents to switch specialties in the middle of training if their career aspirations change, so they can spend the majority of time in their chosen specialty. This possibly could be accomplished if a 3-year core became solidified with the American Board of Surgery.

Offering 2 pathways can be challenging if trainees in each pathway perceive differences in education. At USC, we have graduated 2 traditional trainees after inception of our integrated program and have matched 2 more for this upcoming academic year. As mentioned above, I believe that both pathways can produce excellent surgeons, and the culture of education far outweighs which pathway a candidate has chosen. I ensure that the final 3 years of our I-6 program are identical in structure to our traditional 3-year program. In fact, there is no separation in rotations or experience for any resident in the final 3 years of training.

There are potential challenges in accepting younger learners into our specialty. Despite a strong perceived interest and commitment to thoracic surgery, the attrition rate will likely be higher from medical school applicants compared with those who apply during general surgery training. I do not have data on national dropout rates, but it certainly has been part of the national discussion and concerns about I-6 programs. To date, we have had 3 trainees leave the program, all during the first 3 years. The reasons for this were varied. Two residents left after PGY-2, one for unsuspected medical reasons related to refractory tendinitis and another to pursue a nonmedical career. The third resident left after numerous academic warnings and probation, acknowledging the inability to render safe and appropriate patient care and unsuitability for our specialty. Attrition for whatever reason can be extremely difficult, and programs need to be prepared for this unsuspecting occurrence. It likely can be minimized with refinements and reflections of an institutions interview and selection process but likely will not be eliminated. We were able to make some short-term changes to our rotation assignments, but ultimately matched into our existing traditional program to fill the vacancies. Accepting candidates into our traditional program also nullified the vacancy created at the inception of the research year, which was required of all residents matriculating after 2014. As can be seen, having more than a single pathway can be advantageous for an institution if the educational commitment and experience to the senior learners in each program are similar. Obviously, there is more work in maintaining certification for different programs, but I believe the benefits outweigh the disadvantages provided that trainees can coexist without any perceived differences in commitment to their education.

In the past, integrated programs have attracted mainly “cardiac” tract candidates. However, an integrated curriculum should be flexible and capable of training both cardiac and general thoracic pathways. Six years of clinical training is ample time to build in early rotations in robotic surgery, interventional bronchoscopy, advanced laparoscopy, and other skills necessary for advanced training in general thoracic surgery. This likely will require existing I-6 programs to offer different tracks and require uncommitted candidates to choose a track by the end of second or third year. In thinking about our program, I would establish a certain number of core rotations completed by each track with an increased number of cardiac or thoracic electives/rotations that would be track-specific.

Designing an ideal training program mandates that we consider what is in the best interest of the trainee. This is difficult, as curricula often reflect the needs of a given practice or institution. Allowing flexibility within training programs would require increased resources, including additional residency positions. Most programs perceive they have the bare number of residents just “to get the work done.” I believe that this situation stifles educational innovation and the ability to make substantial changes to long-standing curricula.

The ideal training program in thoracic surgery should include 6 to 7 years of clinical training after medical school. This allows for the majority of postgraduate clinical training to occur in thoracic surgery. I believe this pathway more adequately prepares trainees for independent practice than current traditional paradigms. The development and approval of integrated programs provided an important step forward in the training of thoracic surgeons. Integrated programs should permit substantial exposure to thoracic surgery during the first 3 years of training. Technologic advances and emerging innovation will require us to maintain flexibility in our training paradigms.

### Conflict of Interest Statement

The author reported no conflicts of interest.

The *Journal* policy requires editors and reviewers to disclose conflicts of interest and to decline handling or reviewing manuscripts for which they may have a conflict of interest. The editors and reviewers of this article have no conflicts of interest.
